# First identification and multilocus genotyping of *Giardia duodenalis* in pet chipmunks (*Eutamias asiaticus*) in Sichuan Province, southwestern China

**DOI:** 10.1186/s13071-018-2790-z

**Published:** 2018-03-20

**Authors:** Lei Deng, Run Luo, Haifeng Liu, Ziyao Zhou, Lingyu Li, Yijun Chai, Leli Yang, Wuyou Wang, Hualin Fu, Zhijun Zhong, Suizhong Cao, Guangneng Peng

**Affiliations:** The Key Laboratory of Animal Disease and Human Health of Sichuan Province, College of Veterinary Medicine, Sichuan Agricultural University, Chengdu, Sichuan Province 611130 China

**Keywords:** *Giardia duodenalis*, Chipmunk, Assemblage, Multilocus genotype

## Abstract

**Background:**

*Giardia duodenalis* is a flagellated parasite that causes diarrhea in humans and other animals. Although *G. duodenalis* is found in companion animals worldwide, information regarding the prevalence and genetic characteristics of *G. duodenalis* in pet chipmunks in China is limited. The present study therefore aimed to investigate the prevalence and genotypes of *G. duodenalis* in pet chipmunks in Sichuan province, southwestern China, as well as to assess zoonotic potential of revealed assemblages.

**Results:**

A total of 279 fecal samples were collected from pet chipmunks in seven pet shops and one breeding facility in Sichuan province, southwestern China. The prevalence of *G. duodenalis* was 8.6% (24/279), as determined by nested PCR detection of the beta giardin (*bg*) gene. *Giardia duodenalis* assemblages and subtypes were determined using multilocus genotyping of the *bg*, triosephosphate isomerase (*tpi*), and glutamate dehydrogenase (*gdh*) loci. Two assemblages were identified: potentially zoonotic assemblage A (54.2%, 13/24) and rodent-specific assemblage G (45.8%, 11/24). A total of 24, 17 and 17 sequences of the *bg*, *gdh* and *tpi* loci, respectively, were successfully obtained, which formed four, four and three subtypes, respectively. Moreover, four assemblage A (MLGs A1-A4) and three assemblage G (MLGs G1-G3) multilocus genotypes were identified.

**Conclusions:**

To our knowledge, this is the first study that investigated *G. duodenalis* in pet chipmunks in China. Detection of assemblage A in pet chipmunks and in previous studies in humans suggests a possible role of chipmunks as a reservoir for human giardiasis in Sichuan Province, China.

**Electronic supplementary material:**

The online version of this article (10.1186/s13071-018-2790-z) contains supplementary material, which is available to authorized users.

## Background

*Giardia duodenalis* is a common enteric parasite that infects a broad spectrum of vertebrate hosts, including humans, livestock, companion animals and wildlife worldwide [1]. Usually, humans and animals become infected with *G. duodenalis via* fecal-oral route by ingesting infective stage cysts in contaminated food or water [2]. Clinical manifestations vary depending upon the immunological status of the host [3]. In immunocompetent individuals, *G. duodenalis* causes self-limiting diarrhea and malabsorption, whereas in infants, elderly persons, and individuals with deficient immune system, it also can cause chronic or life-threatening diarrhea and weight loss [4, 5].

Molecular studies have demonstrated that *G. duodenalis* comprises at least eight genetically distinct assemblages (A-H) [6]. Assemblages A and B, considered zoonotic genotypes, can infect humans and a wide range of mammalian hosts, whereas the remaining assemblages (C-H) seem to be host-specific [7]. Assemblages C and D mainly infect dogs, assemblage E livestock, assemblage F cats, assemblage G rodents, and assemblage H marine mammals [8, 9]. So far, assemblages A and B have been determined as predominant genetic sub-populations of *Giardia* in pet rodents in countries such as Spain, Italy, Australia, Belgium and China [10–12]. In addition, assemblages C, G, and E have been also occasionally identified in pet rodents [12, 13].

In China, epidemiological data about *Giardia* prevalence have been reported in domestic dogs and cats (assemblages C and D) [14], raccoon dogs (assemblages C and D) [15], cattle (A, B, and E) [16], horses (assemblages A and B) [17], sheep, goats (assemblages A, B and E) [18, 19], pet chinchillas (assemblages A and B) [11], as well as in humans (assemblages A and B) [20]. Recently, chipmunks (*Eutamias asiaticus*) have become popular in China as companion animals. As such, they have been commercially bred in significant numbers in many breeding facilities. However, information regarding *G. duodenalis* infection in pet chipmunks in China is rare, and the zoonotic potential of this parasite in chipmunks remains unknown. Therefore, the purpose of this study was to identify the prevalence and assemblages of *G. duodenalis* in pet chipmunks and to determine the genetic characteristics of *G. duodenalis*-positive samples.

## Methods

### Specimen collection

From March 2016 to April 2017, 279 fecal specimens were collected from chipmunks in seven pet shops and one breeding facility located in Sichuan province, southwestern China (Additional file 1: Table S1). Chipmunks were bred in individual cages. Fecal samples were collected from the bottom of the each cage, immediately placed into individual 30 ml sterile containers, and taken to the laboratory in a cooler with ice packs within 24 h. At sampling time, the age, sex, and health condition were recorded. All chipmunks whose fecal samples were examined were in apparently good health at the time of sampling.

### DNA extraction and PCR amplification

All fecal specimens were washed three times by centrifugation at 1500× *g* for 10 min with distilled water. Genomic DNA was extracted from approximately 200 mg of each processed fecal specimen using an E.Z.N.A.R® Stool DNA kit (Omega Biotek Inc., Norcross, GA, USA) according to the manufacturer’s instructions. Extracted DNA was stored at -20 °C until molecular analysis.

*Giardia duodenalis* prevalence and assemblages were first determined by nested PCR amplification of approximately 550 bp fragment of the beta giardin (*bg*) gene. Furthermore, *bg*-positive specimens were also analyzed by PCR amplification of the glutamate dehydrogenase (*gdh*) and triosephosphate isomerase (*tpi*) genes. PCR amplification primers and annealing temperatures in this study (Table 1) were as previously reported [21]. PCR products were visualized by electrophoresis in 1.5% agarose containing ethidium bromide.

**Table 1:** Primer sequences, annealing temperatures and the fragment lengths of the genes used in this study

Gene	Primer	Sequence (5'-3')	Annealing temperature (°C)	Fragment length (bp)	Reference
*bg*	F1	AAGCCCGACGACCTCACCCGCAGTGC	65	530	[21]
	R1	GAGGCCGCCCTGGATCTTCGAGACGAC			
	F2	GAGGCCGCCCTGGATCTTCGAGACGAC	65		
	R2	CATAACGACGCCATCGCGGCTCTCAGGAA			
*tpi*	F1	AAATIATGCCTGCTCGTCG	50	530	[21]
	R1	CAAACCTTITCCGCAAACC			
	F2	CCCTTCATCGGIGGTAACTT	50		
	R2	GTGGCCACCACICCCGTGCC			
*gdh*	F1	TTCCGTRTYCAGTACAACTC	50	511	[21]
	R1	ACCTCGTTCTGRGTGGCGCA			
	F2	ATGACYGAGCTYCAGAGGCACGT	50		
	R2	GTGGCGCARGGCATGATGCA			

### Sequence and phylogenetic analyses

All amplified products were sent to Life Technologies (Guangzhou, China) for sequencing on an ABI 3730 DNA Analyzer (Applied Biosystems, Foster City, CA, USA) using the BigDye® Terminator v3.1 cycle sequencing kit. Nucleotide sequence accuracy was confirmed by sequencing two separate PCR products. Assemblages and subtypes were identified by aligning nucleotide sequences with known reference *tpi*, *gdh* and *bg* gene sequences of *G. duodenalis* available in GenBank using BLAST (http://www.ncbi.nlm.nih.gov/BLAST/).

A phylogenetic analysis was performed by constructing a neighbor-joining tree using Mega 6 software [12], based on the evolutionary distances calculated by a Kimura 2-parameter model. The reliability of these trees was assessed using bootstrap analysis with 1000 replicates.

## Results and discussion

The present study identified 24 (8.6%, 95% CI: 0.053–0.119%) fecal samples that were *G. duodenalis*-positive by PCR analysis of the *bg* gene. In China, the prevalence of giardiasis varies from 1.9% [22] to 27.1% [11] in pets by PCRs. In Heilongjiang Province, the rates of infection with *G. duodenalis* in pet cats and dogs were 1.9 and 4.5%, respectively [22]. In Guangzhou, 11% of fecal samples form pet dogs were positive for *G. duodenalis* [23]. The prevalence of *G. duodenalis* in pet dogs in Henan Province was 12.4% [14]. In Niaoning Province, the infection rate of *G. duodenalis* in farm dogs was 13.2% [24]. In Shanghai, Hualiu et al. [25] reported that *G. duodenalis* was found in 26.67 and 9.26% of samples from pets and zoo animals, respectively. The infection rate of *G. duodenalis* in pet chinchillas was 27.1% in four different cities in China [11]. The variability of *G. duodenalis* prevalence in China may be explained by the differences in geographical location, climate, age and gender of the animals, management system, and the diagnostic method.

The genetic diversity of *G. duodenalis*-positive samples was observed by sequencing the *bg*, *tpi* and *gdh* genes. A total of 24, 17 and 17 sequences were obtained for the three gene loci, respectively (Table 2). Analysis of the *bg* gene identified 13 sequences as belonging to assemblage A (9 subtype AI and 4 subtype AI-1) and 11 to assemblage G (7 subtype GI and 4 subtype GII). The subtype AI sequence (MF671918) was identical to the known assemblage AI sequences: accession no. KR051224 (from tortoise in China) and accession no. GQ329671 (from human in Sweden [26]). The subtype AI-1 sequence (MF671917) had one single nucleotide polymorphism (SNP) when compared to sequence KR051224 (Table 3). The subtype GI sequence (MF671919) was identical to sequence EU769221 (from *Rattus norvegicus* in Sweden), whereas the subtype GII sequence had two SNPs (at position 97 G/A, and at position 325 A/C) in comparison with sequence EU769221. Among 17 samples positive for the *tpi* gene, 13 sequences were identified as assemblage A (5 subtype AI and 8 subtype AV) and 4 were identified as assemblage G (2 subtype GI and 2 subtype GII). The genetic characterization is presented in Table 3. Subtype GII sequence (MF671913) was identical to sequence EU781013 (from *Rattus norvegicus* in Sweden), whereas subtype GI sequence had one SNP (at position 184 C/T) in comparison with sequence EU781013. Out of 17 samples positive for the *gdh* gene, 10 sequences were identified as belonging to assemblage A (7 subtype AI and 3 novel subtypes AI-1) and 7 were identified as belonging to assemblage G. The novel subtype AI-1 sequence had not been reported previously; it had 5 SNPs when compared with sequence KR075940 (from sheep in China [27]) (Table 3). Assemblage G sequence was identical to sequence AY178748 (from mouse in Australia). The phylogenetic trees based on *bg*, *gdh* and *tpi* are presented in Additional file 2: Figure S1.

**Table 2:** Assemblages of *Giardia duodenalis* and the distribution of *bg*, *tpi* and *gdh* sequences for each positive samples and multilocus characterization

Chipmunk ID	Assemblage			GenBank ID	MLG type (*n*)
	*bg*	*tpi*	*gdh*		
CD26, CD27	GI	GI	G	MF671919, MF671914, MF671912	MLG G1 (2)
CD28	GI	GII	G	MF671919, MF671913, MF671912	MLG G2 (1)
CD79	GII	GII	G	MF671920, MF671913, MF671912	MLG G3 (1)
CD29, CD34, CD69	AI	AV	AI-1	MF671918, MF671915, MF671910	MLG A1 (3)
CD60, CD119	AI	AI	AI	MF671918, MF671916, MF671911	MLG A2 (2)
CD61, CD96, CD124, CD144	AI-1	AV	AI	MF671917, MF671915, MF671911	MLG A3 (4)
CD64	AI	AV	AI	MF671918, MF671915, MF671911	MLG A4 (1)
CD74, CD92, CD186	AI	AI	Neg	MF671918, MF671916, –	–
CD81, CD203, CD211	GII	Neg	Neg	MF671920, –, –	–
CD22, CD44, CD102	GI	Neg	G	MF671919, –, MF671912	–
CD23	GI	Neg	Neg	MF671919, –, –	–

*Abbreviations*: *Neg* no genotype determined; – GenBank accession numbers not available

**Table 3:** Variations in the *bg*, *tpi* and *gdh* genes among *Giardia duodenalis* assemblage A isolates from chipmunks in Sichuan Province, southwestern China

Subtype	GenBank ID	Gene	Nucleotide at position					
			74	248	275	287	443	469
AI (Ref.)	KR051224	*bg*	A	A	C	A	A	T
AI	MF671918	*bg*	A	A	C	A	A	T
AI-1	MF671917	*bg*	A	A	C	C	A	T
AI (Ref.)	EU014517	*tpi*	C	A	A	A	C	G
AI	MF671916	*tpi*	C	A	A	A	C	G
AV (Ref.)	KP780973	*tpi*	C	T	C	G	A	T
AV	MF671915	*tpi*	C	T	C	G	A	T
AI (Ref.)	KR075940	*gdh*	C	T	G	G	C	A
AI	MF671911	*gdh*	C	T	G	G	C	A
AI-1	MF671910	*gdh*	T	C	A	G	T	C

In the present study, assemblage A sequences were more prevalent than assemblage G sequences, and no mixed assemblage A and G infections were detected. This result is consistent with data from previous studies in pets in China and Mexico [24, 28]. Thus far, assemblages A-C, E and G have been identified in rodents worldwide, and nearly all tested samples contained at least either assemblage A or B [12, 13]. Subtyping assemblage A sequences at different loci has revealed three sub-assemblages: AI, AII, and AIII. In China, sub-assemblage AI has been detected in a variety of hosts, including humans, cattle, goat, sheep, dogs, cats and pigs [7]. Sub-assemblage AII has also been identified in humans in China. Cross-species transmission of *G. duodenalis* assemblage AI in Mexico and in Brazil has been recently reported [28, 29]. This finding suggests that chipmunks infected with assemblage AI were probably the source of human giardiasis due to frequent contact of the two species in that specific area. However, the generalization of this proposal requires systematic, molecular epidemiological investigations in humans and animals in other geographical areas. Recently, the multilocus genotype (MLG) model has been widely used to better understand the characteristics of *G. duodenalis* in different hosts and to assess its zoonotic potential [30, 31]. In this study, 14 samples were successfully sequenced at all three gene loci, and were characterized as four assemblage A MLGs and three different assemblage G MLGs (Table 2).

## Conclusions

In the present study, for the first time, we described the rates of infection with *G. duodenalis* of pet chipmunks in Sichuan province of southwestern China and demonstrated the prevalence of assemblages A and G. On the basis of the multilocus sequence analysis, four assemblage A MLGs and three assemblage G MLGs were identified. Because assemblage A *G. duodenalis* is known to be zoonotic and has been identified in humans in China, pet chipmunks infected with assemblage A constitute a potential zoonotic risk to humans.

## Additional files

Additional file 1: **Table S1.** Detailed information about each chipmunk. (XLSX 20 kb)

Additional file 2: **Figure S1.** Phylogenetic relationships of *Giardia duodenalis* for the *bg*, *tpi*, and *gdh* loci (**a**, *bg*; **b**, *tpi*; **c**, *gdh*). The relationships between *G. duodenalis* genotypes identified in this study and other known genotypes deposited in GenBank were inferred by a neighbor-joining analysis of three genetic loci using the Kimura 2-parameter model. Bootstrap values greater than 50% from 1000 replicates are shown. Sequences obtained in this study are marked with a triangle. (TIFF 529 kb)

## Abbreviations

*bg*: β-giardin
*tpi*: Triosephosphate isomerase
*gdh*: Glutamate dehydrogenase
MLGs: Multilocus genotype
SNP: Single nucleotide polymorphism
