# Supplementary figures and images for: First identification and multilocus genotyping of Giardia duodenalis in pet chipmunks (Eutamias asiaticus) in Sichuan Province, southwestern China
Source: Parasit Vectors. 2018 Mar 20;11:199. doi: 10.1186/s13071-018-2790-z (PMC5859636; doi:10.1186/s13071-018-2790-z)

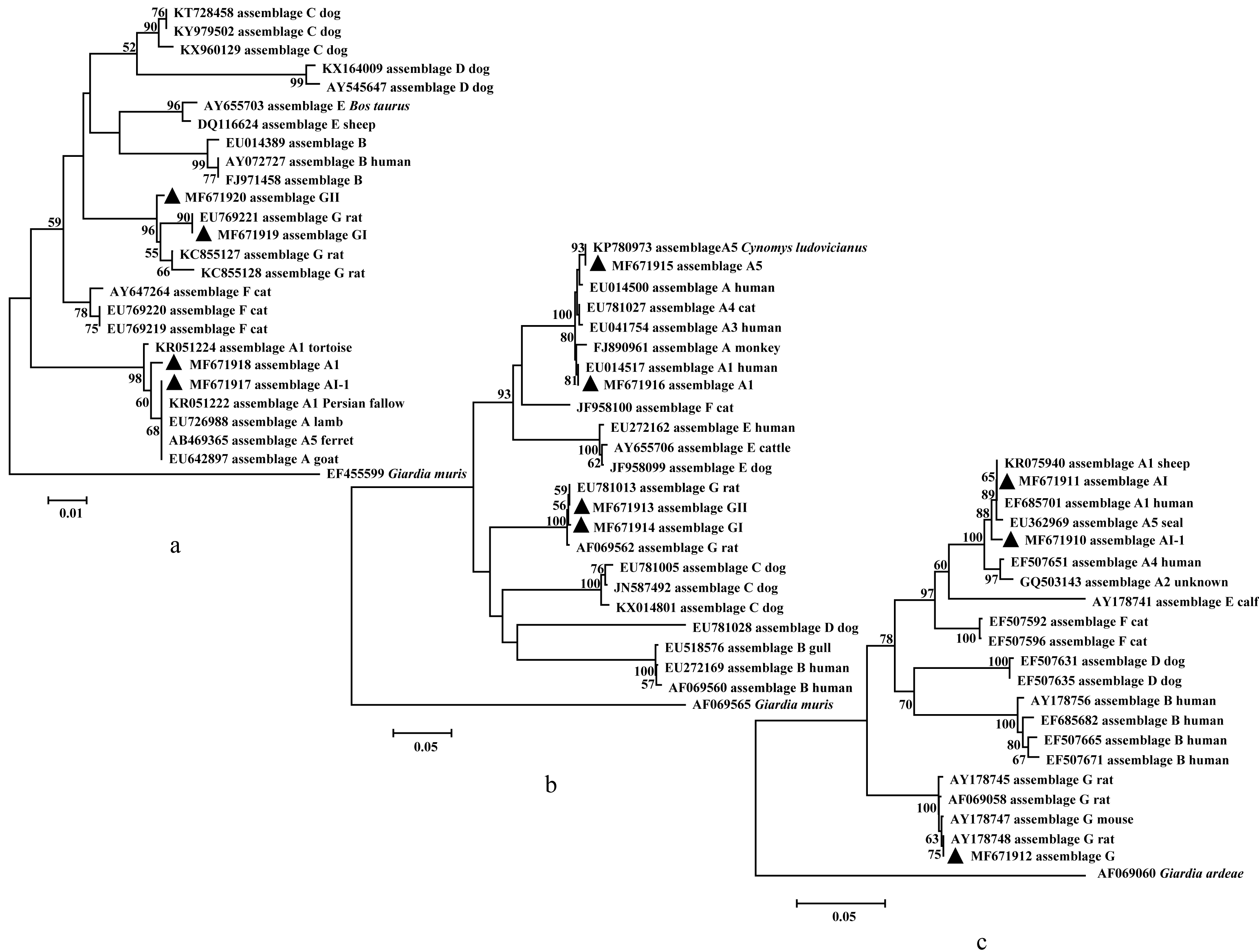

Supplement: Supplementary file 2 — Figure S1. Phylogenetic relationships of Giardia duodenalis for the bg, tpi, and gdh loci (a, bg; b, tpi; c, gdh). The relationships between G. duodenalis genotypes identified in this study and other known genotypes deposited in GenBank were inferred by a neighbor-joining analysis of three genetic loci using the Kimura 2-parameter model. Bootstrap values greater than 50% from 1000 replicates are shown. Sequences obtained in this study are marked with a triangle. (TIFF 529 kb) [file 13071_2018_2790_MOESM2_ESM.tif]
